# Pet attachment and owner personality

**DOI:** 10.3389/fpsyt.2024.1406590

**Published:** 2024-04-26

**Authors:** Deborah L. Wells, Kathryn R. Treacy

**Affiliations:** Animal Behaviour Centre, School of Psychology, Queen’s University Belfast, Belfast, Northern Ireland, United Kingdom

**Keywords:** attachment, big five, companion animals, dark triad, human-animal bond, mental health, personality, pets

## Abstract

**Introduction:**

Research points to a relationship between owner personality and strength of attachment to one’s pet, with implications for psychological health. So far, studies in this area, albeit sparse, have focused on the ‘Big Five’ traits of owner personality. The ‘Dark Triad’ is a cluster of traits that has also been linked to emotional deficits, but has been overlooked in relation to pet attachment. This study therefore examined the association between owner personality and pet attachment, focusing on both the ‘Big Five’ and ‘Dark Triad’ traits of personality.

**Methods:**

A cross-sectional design was employed to collect quantitative data from dog and cat owners across the globe between May-June 2023. A purpose-designed online survey collected sociodemographic details, along with information on pet ownership, strength of the pet-owner bond and participant personality, assessed using the Big Five personality scale and the Short Dark Triad scale. The survey was fully completed by 759 dog and 179 cat owners.

**Results:**

Analysis revealed significant correlations between many of the participants’ personality traits, both within and between scales. Strength of pet attachment was positively correlated with neuroticism and conscientiousness, and, more weakly, to Machiavellianism. Regression analysis revealed that females, dog owners, people over the age of 50 and individuals who had children under 18 years to care for were more strongly attached to their pets than others. Both neuroticism and conscientiousness were found to be significant predictors of participants’ pet attachment scores. None of the Dark Triad traits significantly predicted the criterion.

**Discussion:**

This study points to a relationship between strength of attachment to one’s pet and owner personality, at least as measured using the Big Five approach to personality assessment. There was little to support an association between the Dark Triad traits and strength of attachment to one’s pet, although the link between these characteristics and attachment styles is still unknown. The investigation lends support for the idea that high attachment levels are associated with personality traits aligned to psychological ill-health. Further work is recommended in this area, with a greater focus on both strength and quality (e.g., attachment style) of the pet-owner bond.

## 1 Introduction

Pet ownership is a global phenomenon in today’s society, with over 500 million pets residing in homes across the world (1). Figures show that in the United Kingdom alone, over 12 million dogs and 11 million cats were kept as pets in 2023, with incidence figures increasing on a yearly basis (2).

Although people keep pets for a myriad of reasons (e.g., companionship, recreation, protection), some acquire a companion animal in the belief that it offers health advantages [for reviews see (3, 4)]. Numerous studies have explored the widely held claim that ‘pets are good for us’, with some yielding positive results in this respect, particularly in relation to dog ownership [for review see (5)]. For example, pet ownership has been found to be negatively associated with depression in homeless youths (6), men infected with AIDS (7) and dog-owners living with HIV (8). The ownership of a pet, and again notably a dog, may also have a role to play in improving cardiovascular health, perhaps partly because of the increased exercise that typically accompanies the ownership of this species (9, 10). Whilst positive findings are widely published in the area, research does present a somewhat mixed picture, with some studies yielding either null results or pointing to some detrimental associations (11, 12). Amiot and colleagues (13), for instance, reported poorer mental health in pet owners than non-owners during the COVID-19 pandemic, while older Canadian pet owners were found to be less satisfied with their lives than non-pet owners (14).

One factor that may influence the extent to which an owner gains health benefits from their pet is the strength of the human-animal bond. Attachment theory was first proposed by Bowlby (15) to outline the child-caregiver relationship, but has since been used successfully to explain owner-pet relationships (16, 17), with studies suggesting that companion animals can serve as important attachment figures (18). One might expect a stronger attachment to one’s companion animal to be associated with enhanced wellbeing, and, indeed, this is supported by some studies. Garrity and colleagues (19), for example, found lower levels of depression in older adults who reported higher attachment to their pets than more weakly bonded individuals. More recently, Teo and Thomas (20) reported that people who were “securely” attached to the animals in their care had lower levels of psychological distress and psychopathology and better quality of life than individuals less securely attached. Whilst perhaps counter-intuitive, some studies in this area have reported poorer mental health in people who are more strongly bonded with their pets. Wells and colleagues (21), for instance, found that higher bonds of attachment to one’s dog or cat were associated with higher levels of depression, loneliness and lower levels of positive experience. Miltiades and Shearer (22) likewise found that higher levels of attachment to one’s companion animal were associated with higher levels of depression in a group of older American adults, while Lass-Hennemann and associates (23) reported an association between stronger attachment to one’s dog and higher levels of psychopathological symptoms. One explanation for these discrepant findings may lie with owner personality. Bagley and Gonsman (24), for example, found that people with ‘Idealist’ personality types had significantly higher pet attachment scores than ‘Rationals’ and ‘Artisans’. Reevy and Delgado (25) likewise found a positive correlation between attachment to one’s pet and neuroticism, a personality trait that has been linked to psychological health disorders, notably depression and anxiety (26–28). More recently, a study involving over 2,500 Finnish dog and cat owners reported that neuroticism and poor mental health are linked to ‘anxious’ attachment styles and highlighted the significance of individual personality traits in contributing to insecure attachment and, more generally, mental well-being (29).

So far, research exploring the association between owner personality and pet attachment has focused heavily on the ‘Big Five’ traits [openness to experience, extraversion, neuroticism, conscientiousness, agreeableness, (30)]. Different psychometric tests, however, measure different personality constructs and vary in their utility depending on the criterion under scrutiny (31). Other dimensions of personality are certainly worth focusing on, particularly those, like the ‘Big Five’, known to be linked to mental health. The following study therefore aimed to further explore the link between owner personality and pet attachment, focusing on both the Big Five traits of personality, and the ‘Dark Triad’, a cluster of traits [(Machievellianism, narcissism, psychopathy, (32)] that has been linked to emotional deficits (33) and has been sorely overlooked in relation to pet ownership and attachment. It was anticipated that the work would shed useful light on the link between owner personality and pet attachment, with important implications for its role in psychological well-being.

## 2 Materials and methods

### 2.1 Sampling and participants

Adult dog and cat owners from across the globe were invited to take part in this study via advertisements placed on social media platforms, e.g., Facebook, Twitter, Reddit. The advertisement indicated that the study was concerned with exploring pet-owner relationships, rather than drawing specific attention to its focus on attachment and personality assessment. The online survey attracted a total of 1487 responses. Following screening for inclusion criteria (provision of informed consent, dog/cat ownership, primary pet caregiver, aged 18+ years, proficiency in English) and quality of data (i.e., failure to complete the survey), 549 individuals were removed; the final dataset therefore comprised 938 eligible participants (for full details see Results).

### 2.2 Survey

A purpose-designed questionnaire was developed that aimed to collect information on sociodemographic background, pet ownership status, strength of the pet-owner bond and participant personality. Section 1 of the survey collected demographic information, including details on the respondents’ gender (men, women, other), age (18-35, 36-50, 51+ years), geographic location (UK/Ireland, Americas, Europe, Oceania, Rest of World), marital status (single, married/co-habiting, separated/divorced, widowed) and whether or not they cared for a child under 18 years of age (yes, no). This part of the survey also collected information on pet ownership. Respondents were required to indicate whether they owned a pet dog (yes, no) or cat (yes, no). If individuals owned more than one pet, they were asked to specify which animal (dog or cat) they would focus on for the survey. The survey also collected information on how long individuals had owned their pet (< 1 year, 1-5 years, >5 years).

The second part of the survey collected information on the participants’ personality. Two validated psychometric tests were used:

i) *Big Five Personality Scale-Short* [BFI-S, (34)]. This is a 15-item questionnaire used to measure 5 aspects of personality (openness, conscientiousness, extraversion, agreeableness, and neuroticism). Participants are required to respond to a series of statements (e.g., “I see myself as someone who worries a lot”) using a Likert scale, ranging from 1 (strongly disagree) to 5 (strongly agree). The scale has been shown to have good overall validity (34, 35).

ii) *Short Dark Triad* [SD3, (36)]. The SD3 is a 27-item questionnaire used to measure the ‘Dark Triad’ of personality traits (Machiavellianism, narcissism, and psychopathology). Respondents are asked to indicate their level of agreement with a series of statements (e.g., “It’s not wise to share your secrets”), using a Likert scale ranging from 1 ‘strongly disagree to 5 ‘strongly agree’. The SD3 has good reliability and validity (36).

The final section of the survey (Section 3) collected information on owner-pet attachment. Participants were required to complete the *Lexington Attachment to Pets Scale* [*LAPS*, (37)], a test designed to determine the strength of the animal-owner bond. The LAPS requires owners to assess their degree of agreement with 23 statements (e.g., “I consider my pet to be a friend”) on a 4-point Likert scale, ranging from 0 (strongly disagree) to 3 (strongly agree). The scale has been shown to have good internal consistency (coefficient alpha=0.928) and examines emotional attachment to both dogs and cats. The survey is one of the most commonly used indicators of owner-pet attachment in studies of the human-animal bond (21, 24, 25, 38).

### 2.3 Procedure

Pet owners interested in taking part in the study followed a link to the questionnaire hosted on the online platform Qualtrics. Here, they initially read the Participant Information Sheet, which gave details on what the study entailed. If still keen to take part in the investigation, participants indicated their consent by checking a box and commenced the survey. Individuals who did not meet the necessary inclusion criteria (see earlier) were not allowed to complete the consent form or go any further with the study. Following survey completion, participants were thanked for their time and allowed to read a debrief. The study remained open for one month between May-June 2023.

### 2.4 Data analysis

Simple descriptive statistics were initially carried out to explore the frequency and percentage of responses to the sociodemographic information. Pearson’s moment correlations were subsequently conducted to assess any significant relationships between participants’ personality trait scores (openness, conscientiousness, extraversion, agreeableness, neuroticism, Machiavellianism, narcissism, psychopathology), both within and between scales, and to explore for any associations with their attachment to pet (LAPS) scores. Finally, a linear regression analysis was conducted to examine whether any of the demographic variables or personality traits served as predictors of the strength of pet attachment. Overall LAPS score was set as the criterion variable, while factors of owner gender (men, women [none of the participants checked the ‘other’ category]), age (18-35, 36-50, 51+ years), geographic location (UK/Ireland, Americas, Europe, Oceania, Rest of World), marital status (single, married/co-habiting, separated/divorced, widowed), parental status (parent of child under 18, not parent of child under 18), pet type (dog, cat), length of pet ownership (<1year, 1-5 years, >5 years) and personality traits (openness, conscientiousness, etc.), were set as the predictor variables. The assumptions underlying regression analysis were sufficiently met. Inspection of scatterplots for the continuous predictors revealed linear relationships with the criterion variable. There was no evidence of any multicollinearity between the predictor variables (all variance inflation factor (VIF) values < 1.6; mean VIF=1.32, SD=016). Scatterplots revealed homoscedasticity of residuals, while Q-Q plots showed that the residuals followed a normal distribution.

### 2.5 Ethics

Full ethical approval for the study was granted by the University’s Faculty Ethics Research Committee (EPS 23_174).

## 3 Results

### 3.1 Participants

Demographic information on the participants involved in the study can be found in **Table 1**. As can be seen, most of the participants were from the Global North (UK/Ireland, Europe or the Americas). The majority of respondents were women, under 50 years of age and were married or cohabiting. Just over half of the sample were parents to children under 18 years of age. The vast majority of the cohort reported owning a dog, with most people having cared for their pet for over one year.

**Table 1:** Number and percentage of participants according to demographic factor (*n=938*).

Demographic Factor	N	%
Gender		
Men	139	14.8
Women	799	85.2
Age (years)		
18-35	322	34.3
36-50	335	35.7
51+	281	30.0
Geographic location		
UK/Ireland	285	30.4
Americas	246	26.2
Europe	202	21.5
Oceania	108	11.5
Rest of World	97	10.4
Marital status		
Single	159	17.0
Married/cohabiting	715	76.1
Separated/divorced	54	5.8
Widowed	10	1.1
Parental Status		
Child < 18 years	484	51.6
No child <18 years	454	48.4
Pet ownership		
Dog	759	80.9
Cat	179	19.1
Length of pet ownership		
< 1 year	79	8.5
1-5 years	474	50.5
>5 years	385	41.0

### 3.2 Pet owner personality

Mean personality scores for both the BFI and Dark Triad scales are presented in **Table 2**. Analysis revealed a number of small, although statistically significant, correlations between many of the participants’ personality traits (**Table 3**). All of the Dark Triad traits were positively correlated with each other. Significant correlations were also found between many of the BFI trait scores. Specifically, neuroticism was negatively correlated with traits of extraversion, agreeableness and conscientiousness, while extraversion was found to be positively associated with openness and conscientiousness. Both openness and agreeableness were positively correlated with conscientiousness. A number of Dark Triad trait scores were significantly correlated with BFI scores. Machiavellianism was positively correlated with neuroticism, but negatively associated with traits of conscientiousness, extraversion and agreeableness. Narcissism was positively correlated with openness, conscientiousness and extraversion, but negatively associated with neuroticism. Finally, significant negative correlations were found between psychopathy and BFI traits of conscientiousness and agreeableness.

**Table 2:** Mean (SD) personality scale scores (*n=938*).

Personality Trait	Mean	SD
BFI		
Openness	4.20	0.81
Conscientiousness	4.01	0.84
Extraversion	3.19	1.21
Agreeableness	4.13	0.79
Neuroticism	3.09	1.10
Short Dark Triad		
Machiavellianism	1.84	0.01
Narcissism	1.69	0.01
Psychopathy	1.13	0.01

**Table 3:** Pearson moment correlations between Big Five, Dark Triad and Lexington Attachment to Pets (LAPS) scores.

Trait	O	C	E	A	N	M	Nar	P	LAPS
**O**	**-**								
**C**	0.08*	**-**							
**E**	0.12***	0.09**	**-**						
**A**	0.02	0.21***	0.07*	**-**					
**N**	-0.04	-0.24***	-0.21***	-0.01***	**-**				
**M**	-0.001	-0.13***	-0.08**	-0.32***	0.12***	**-**			
**Nar**	0.22***	0.12***	0.46***	0.005	-0.23***	0.20***	**-**		
**P**	0.03	-0.14***	0.03	-0.44***	-0.02	0.41***	0.23***	**-**	
**LAPS**	0.04	0.13***	-0.01	0.002	0.11***	0.06*	0.05	0.05	**-**

O, openness to experience; C, conscientiousness; E, extraversion; A, agreeableness; N, neuroticism; M, Machiavellianism; Nar, narcissism; P, psychopathy; LAPS, Lexington Attachment to Pets.

*P<0.05; **P<0.01; ***P<0.001.

### 3.3 Personality and pet owner attachment

Three personality traits were significantly correlated with participants’ LAPS scores, all in a positive direction: neuroticism, conscientiousness and, more weakly, Machiavellianism. None of the other personality traits were associated with owners’ strength of attachment to their pets scores (**Table 3**).

A total of 938 cases were analysed for the linear regression model concerned with attachment level, which was found to be significantly reliable (R^2^ = 0.37, F[22,937]=6.78, P<0.001). Gender, age, parental status and pet ownership status all served as significant predictors of participants’ LAPS scores (**Tables 4**, **5**). Women had significantly higher LAPs scores than men, respondents over the age of 50 years were more strongly attached to their pets than younger individuals, carers of children under the age of 18 years had higher scores than individuals without children in this age group, while dog owners were more strongly attached to their pets than cat owners.

**Table 4:** Results of the linear regression analysis for Lexington Attachment to Pets Scale scores involving predictor variables of participant gender, age, geographic location, marital status, parental status, type of pet owned, length of pet ownership, BFI and Dark Triad traits.

Predictor	Standardized β	95% CI	t	P
Gender				
Men (ref)				
Women	0.13	1.42-4.22	3.66	<0.001
Age (years)				
18-35 (ref)				
36-50	-0.02	-1.51-0.86	-0.55	0.58
51+	-0.13	-3.59- -0.94	-3.35	<0.001
Geographic location				
UK/Ireland (ref)				
Americas	0.01	-1.39-1.77	0.24	0.81
Europe	0.07	-1.31-4.12	1.01	0.31
Oceania	0.13	-0.11-6.56	1.89	0.06
Rest of World	0.12	-0.39-6.42	1.73	0.08
Marital status				
Single (ref)				
Married/cohabiting	0.009	-1.20-1.52	0.23	0.81
Separated/divorced	0.05	-0.71-4.07	1.38	0.17
Widowed	0.06	-0.43-9.28	1.79	0.07
Parental status				
No child <18 years (ref)				
Child <18 years	0.18	1.75-3.87	5.21	<0.001
Pet ownership				
Cat (ref)				
Dog	0.20	2.86-5.35	6.47	<0.001
Length of pet ownership				
<1 year (ref)				
1-5 years	0.11	-0.07-3.48	1.88	0.06
>5 years	0.10	-0.24-0.07	1.71	0.09
Personality traits				
Openness	0.04	-0.07-0.34	1.31	0.19
Conscientiousness	0.15	0.26-0.67	4.49	<0.001
Extraversion	-0.04	-0.24-0.06	-1.13	0.26
Agreeableness	0.02	-0.17-0.28	0.48	0.63
Neuroticism	0.13	0.16-0.48	3.88	<0.001
Machiavellianism	0.04	-0.04-0.18	1.25	0.21
Narcissism	0.05	-0.04-0.21	1.36	0.17
Psychopathy	0.03	-0.07-0.18	0.90	0.37

**Table 5:** Mean (SD) LAPS scores according to demographic factor.

Demographic Factor	Mean	SD
Gender		
Men	49.49	8.54
Women	51.72	7.71
Age (years)		
18-35	52.24	7.51
36-50	51.00	7.59
51+	50.89	8.52
Geographic location		
UK/Ireland	51.85	7.63
Americas	51.04	7.83
Europe	51.59	7.90
Oceania	51.86	8.23
Rest of World	50.02	8.15
Marital Status		
Single	51.98	7.86
Married/co-habiting	51.15	7.85
Separated/divorced	52.29	8.42
Widowed	54.50	5.42
Parental Status		
Parent of child <18	52.44	7.27
Not parent of child <18	50.27	8.33
Pet ownership		
Dog	52.17	7.55
Cat	48.13	8.38
Length of pet ownership		
<1 year	50.15	8.52
1-5 years	51.69	7.66
> 5 years	51.29	7.98

LAPS score range=19.0-65.0.

Two of the personality traits served as significant, positive predictors of the criterion variable, namely neuroticism and conscientiousness. People higher in these traits were more strongly attached to their pets than individuals lower in these traits.

## 4 Discussion

This paper explored the relationship between strength of the human-animal bond and owner personality, with a focus, for the first time, on the Dark Triad of traits.

The results of this study showed significant, although modest, interrelationships between many of the participants’ personality traits, both within and between scales. All of the Dark Triad traits were positively correlated with each other, a finding that concurs with other published work in this area (32, 39, 40). These close correlations have led some authors to question whether the Dark Triad traits are sufficiently distinct or harbour an element of conceptual redundancy (41); psychopathy, in particular, is considered by some authors to be indistinct from Machiavellianism (42, 43). Others have suggested that we need to broaden our view of dark personality and instead of considering three traits as one construct, we should perhaps contemplate a construct that encompasses a wider range of ‘dark’ characteristics, e.g., perfectionism, spitefulness, greed (44, 45).

Many of the Big Five traits were also found to be significantly correlated with each other, with the direction of these associations largely in line with published work on personality. For example, neuroticism has been found to be robustly negatively correlated with traits of agreeableness, conscientiousness and extraversion, at least at the between-person level of analysis (see (46) for a discussion of this issue), and indeed a negative correlation between these variables was found in the current study. Likewise, as unearthed here, other authors have found a negative correlation between extroversion and neuroticism and a positive association with openness to experience (47).

Significant associations were found between some of the Big Five factors and Dark Triad traits. For example, Machiavellianism and psychopathy were negatively correlated with Big Five traits of conscientiousness and agreeableness. Narcissism, by contrast, was positively correlated with openness to experience, conscientiousness and extraversion, but negatively correlated with neuroticism. Other authors have reported correlations between the Dark Triad and the Big Five variables (48). Whilst findings have been somewhat inconsistent in relation to exactly which traits are correlated and the direction of these relationships, the current investigation largely aligns with this work (32, 40, 49).

The results from this study revealed positive correlations between people’s attachment to pet scores (LAPS) and traits of neuroticism, conscientiousness and, to a weaker degree, Machiavellianism. Some of these personality traits are associated with poor mental health outcomes. Neuroticism, in particular, has been associated with the propensity to experience negative emotions, including sadness, anger, loneliness, anxiety and feelings of vulnerability (50, 51). People who score more highly for this trait are at greater risk from a wide variety of psychological disorders, including obsessive-compulsive disorder (OCD), bipolar disorder, major depression and schizophrenia [for review see (52)]. Machiavellianism, a trait found to be positively correlated with neuroticism in this study, albeit weakly, has also been linked with poorer mental health, reduced happiness, low self-esteem and higher levels of anxiety and depression (53–55). Whilst neuroticism and Machiavellianism could be considered disadvantageous traits from a mental health perspective, conscientiousness, by contrast, has typically been associated with benefits. People who score highly for this trait, for example, tend to have better physical and mental health, stronger relationships and greater longevity [for review see (56)].

The findings from the current investigation concur with previous work regarding the variables that predict strength of the pet-owner bond. Gender was found to be one of the strongest predictors of the criterion variable, with women being more closely attached to their pets than men, a finding that has been widely reported (21, 57, 58) and may be linked to women showing greater levels of empathy (59, 60). The current study also found a significant association between level of pet attachment and parental status, with people who had children under the age of 18 years to care for being more strongly attached to their pets than individuals without these responsibilities. Interestingly, Wells and colleagues (21) reported the opposite relationship to the findings presented here; their study, however, was conducted during a COVID-19 lockdown, when parents of young children were likely to have been busy trying to juggle working from home with homeschooling, perhaps leaving less time to invest in, or bond with, their pets. In accordance with other studies (21, 23, 61–63), dog owners were found to be more strongly attached to their pets than cat owners. This discrepancy in attachment may be related to the social nature of these animals, with dogs developing stronger bonds of attachment, particularly to humans (64, 65), than cats. Unlike other companion animals, dogs are also more likely to respond to human emotions, even adapting their behaviour in response to their carers’ emotional cues, thereby encouraging closer bonds of attachment (66).

Several authors have unearthed a positive correlation between pet owner attachment and poor mental health (21–23), leading one to question whether high attachment levels are associated with personality traits aligned to psychological ill-health. The results from the present investigation lend support for this, although other factors, including type of pet owned and parental status served as stronger predictors of strength of attachment than personality (see above). In relation to personality traits, however, higher levels of neuroticism, a trait known to be associated with poor mental health (see earlier), were associated with higher pet attachment scores. Interestingly, studies that have focused on the *nature* of the pet-owner bond (as opposed to the *strength* of the relationship, explored here) have shown that high levels of neuroticism are positively correlated with an ‘anxious’ style of attachment, i.e., one that reflects having worries about the pet being available, sensitive and/or responsive to the owner’s needs (25, 29, 67). These types of thoughts and expectations have also been reported in inter-human attachments and are deemed somewhat maladaptive working models (68).

Both the present investigation, and other studies (25, 67), also found that conscientiousness positively predicted strength of pet attachment. This personality trait is typically associated with positive mental health outcomes (see earlier). That said, it is still unclear whether this trait is linked to adaptive or maladaptive attachment styles. For example, the trait has been found to be negatively correlated with both ‘anxiety’ and ‘avoidance’ styles of attachment (25, 67), hinting at a more functional type of relationship. However, Stahl and colleagues (29) recently found that more conscientious cat owners were more anxiously attached to their pets. Going forwards, it is recommended that further consideration is given to the potentially important relationship between strength of attachment to one’s pet, attachment style and mental health. The results from both the present study and other investigations in this area show that people with different personality types may have similar strengths of attachment to their pets, but potentially different attachment styles that may differ in terms of their adaptivity.

One might have expected some of the Dark Triad traits to have served as significant predictors of people’s strength of attachment to their pets, particularly considering the correlation (albeit modest) that was unearthed between Machiavellianism and LAPS scores. The Dark Triad has been associated with various indicators of parenting style, with authoritative parenting being negatively correlated with Dark Triad tendencies and authoritarian and detached parenting more positively correlated with these traits (69). Vonk and colleagues (70) also found that people who were high in grandiose narcissism [as assessed by the ‘Pathological Narcissism Inventory, (71)] were more attached to their ‘traditional’ pets (e.g. dog, cat, hamster) than individuals lower in this trait (this correlation was not unearthed for owners of ‘untraditional’ pets, e.g. reptiles, amphibians, parrots). Of interest, the Dark Triad has been associated with both a general dislike of animals and animal cruelty (72); it may therefore be the case that people high on these personality traits are generally less likely to own animals, or to own them for different reasons, than individuals lower on these traits. Indeed, it has been argued that people with dark personalities may be more inclined to own exotic species [for financial gain and status, (73, 74)], animals not of focus in the current investigation. Of note, lower mean scores for all of the Dark Triad traits were found in the present study compared to other populations (36, 75); again, this could lend some support for the idea that people high in these traits are less likely to own pets and may also explain the lack of significant associations unearthed here (i.e., a floor effect).

Owner personality has important implications, not only for their own health, but that of their pets. Reevy and Delgado (25) found that a high level of neuroticism was associated with a high level of affection towards a pet and high anxious attachment, leading them to argue that neuroticism may offer benefits to a pet’s welfare, with people high on this trait perhaps being more perceptive and responsive to changes in the animal’s behaviour or health. Pet owners prone to this style of attachment do indeed report higher levels of caregiving and attentiveness to their animals (76). The impact of this on the psychological welfare of their animals, however, is very much open to debate. Indeed, neuroticism has been linked to the manifestation of various pet behaviour problems, including destructiveness, sexual mounting and owner-directed aggression (77). Gobbo and Zupan (78) found that dogs of more neurotic caregivers displayed more aggression, both towards conspecifics and humans, while Finka and associates (79) showed a link between higher owner neuroticism and an increased likelihood of cats having a behaviour problem. Together, these studies suggest that neuroticism may be a maladaptive personality trait, both for humans and their pets alike.

## 5 Limitations

Like other studies in this area, there are limitations to this investigation that must be acknowledged. Firstly, it is possible that the online recruitment method employed attracted a certain cohort of people, e.g., individuals who were overly attached to their companion animals. As with most, if not indeed all, studies in this area, the majority of the participants were women, a variable found to be associated with both companion animal attachment and mental well-being. Although challenging, it would be useful for future studies to focus more specifically on men, particularly in light of the difference in attachment styles that exist between the sexes (80, 81). Whilst the scale used to assess pet attachment (LAPS) in the present study is the most commonly employed in this area, a response bias leaning towards higher attachment (perhaps with participants feeling fearful of being perceived as ‘unloving’ of, or ‘unbonded’ to their pets) cannot be ruled out. Future studies may be able to address this by including additional, perhaps more objective, measures of pet attachment (e.g., recording frequency of physical contacts between owners and their pets, oxytocin levels, etc.) and exploring the relationship between these types of attachment tool. Although this study was concerned with exploring the link between pet-owner attachment and owner personality, the role of other variables must be considered. For example, Lass-Hennemann and colleagues (23) found that attachment to humans mediated the relationship between mental health and strength of attachment to one’s dog. Future research needs to consider the wide variety of variables that may be associated with attachments and mental well-being beyond those considered here. Other studies have reported an influence of pet owner race, ethnicity, economic status, etc. (82), and the role of these demographic variables is worth exploring in future statistical models. This study also attracted participants from the global north, rendering it difficult to generalize findings beyond those reported here. Finally, it is worth remembering that this research focused purely on the strength of the owner-pet bond; further work is recommended in this area, with a greater focus on both strength and quality (i.e., attachment styles) of the pet-owner bond.

## 6 Conclusions

Overall, this study points to a relationship between strength of attachment to one’s pet and owner personality, at least as assessed using the Big Five approach to personality measurement. There was little to support the idea that the Dark Triad traits were associated with strength of attachment to one’s pet, although the link between these characteristics and attachment styles is still unknown. There are clearly important links between human-animal attachment and mental health outcomes, both for people and their pets. Developing scales that assess attachment relationships is therefore important from a One Health perspective. There may be benefits to moving beyond the two-dimensional models of human attachment (83) thus far employed in research on owners and their pets. Studies also need to explore, ideally using longitudinal approaches, directionality of attachment bonds and the degree of interdependence between traits of owners and their companion animals. Attachment theory points to a bidirectional relationship, with bonds shaped by both parties (84). Future studies may like to explore direction of causation to more fully understand the complex interactions between human and pet personality traits and the psychological health outcomes for both partners.

## Data availability statement

The datasets presented in this article are not readily available because the dataset presented in this paper is considered confidential as consent for open access was not secured at the time of participant recruitment. Requests to access the datasets should be directed to d.wells@qub.ac.uk.

## Ethics statement

The studies involving humans were approved by Queen’s University Belfast Faculty Ethics Research Committee. The studies were conducted in accordance with the local legislation and institutional requirements. The participants provided their written informed consent to participate in this study.

## Author contributions

DW: Conceptualization, Formal analysis, Methodology, Project administration, Supervision, Validation, Writing – original draft, Writing – review & editing. KT: Data curation, Writing – review & editing.
